# Factors associated with low adherence to cervical cancer follow-up retest among HPV+/ cytology negative women: a study in programmatic context in a low-income population in Argentina

**DOI:** 10.1186/s12885-019-5583-7

**Published:** 2019-04-23

**Authors:** Juan Gago, Melisa Paolino, Silvina Arrossi

**Affiliations:** 1 Programa Nacional de Prevención de Cancer Cervicouterino/Instituto Nacional del Cáncer (Argentina), Julio A. Roca 781, Piso 7, Buenos Aires, Argentina; 2 Centro de Estudios de Estado y Sociedad/Consejo Nacional de Investigaciones Científicas y Técnicas, Sánchez de Bustamante 27, Buenos Aires, Argentina

**Keywords:** HPV, Cervical Cancer, Adherence, Follow-up, HPV test

## Abstract

**Background:**

Cervical Cancer is still a major public health challenge in low and middle-income countries. HPV testing has been an innovative approach, which was introduced in Argentina for women aged 30+ through the Jujuy Demonstration Project (JDP) carried out between 2012 and 2014. After a positive HPV-test, cytology is used as triage method. Under this protocol, the group of women with HPV+ and normal cytology are recommended to repeat the test within 12–18 months. Studies have shown that this group has increased risk of CIN2+, however, assuring high levels of repeating test among these women is difficult to achieve. We analyze those factors associated with lower re-test attendance among HPV+/ cytology negative women at a programmatic level in low-middle income settings.

**Methods:**

We used data of women aged 30+ HPV-tested in the JDP and followed until 2018 (*n* = 49,565). We performed a set of different adjusted logistic regression models.

Primary outcomes were re-test attendance and re-test attendance within recommended timeframe. We assessed as covariates age, health insurance status, year of HPV-testing, Pap testing in the past 3 years, HPV-testing modality (clinician-collected (CC) tests/self-collected (SC) tests), and span between HPV-test collection and report of results.

**Results:**

Forty nine thousand five hundred sixty five women were HPV-tested and 6742 had a positive HPV-test. Among HPV+ women, a total of 4522 were HPV+/Cytology negative (67.1%). In total, 3172 HPV+/Cytology negative women (70.1%) had a record of a second HPV test as of March 2018. Only 1196 women (26%) completed the second test within the timeframe. Women with no record of a previous Pap (OR: 0.46, 95% CI: 0.4–0.53, *p* < 0.001), aged 64+ (OR: 0.46, 95% CI: 0.31-0.68, *p* < 0.001) were less likely to be retested; while women with clinician-collected samples had higher odds of being re-tested (OR: 1.42, 95% CI: 1.06–1.91, *p* < 0.001).

**Conclusions:**

Low re-test rates were found in HPV +/ normal cytology women. Tailored interventions are needed to increase the effectiveness of the screening in this group, especially for those women with characteristics associated to lower attendance.

## Background

Cervical cancer is still a major public health challenge in low and middle-income countries. Unsuccessful screening strategies have been pointed out as one of the major barriers to reduce the morbidity and mortality [1]. Cytology screening presented a particular set of problems, as it required frequent examinations and rigorous quality controls to compensate for its low negative predictive value, low-moderate sensitivity and highly variability depending on the operator [2]. Introduction of HPV testing is an innovative approach in cervical cancer screening programs, which can help to overcome certain limitations of cytology-based schemes. Automated HPV test processing and interpretation reduces the need for cytotechnicians and quality controls. Also, its high sensitivity and negative predictive value allows extension of the screening interval [3, 4]. Furthermore, after the introduction of HPV vaccination, HPV test is recommended as the test for primary screening [2, 5].

Nonetheless, screening strategies based on HPV testing as primary screening have opened new challenges; one is the fact that HPV-testing identifies women positive for HPV but does not inform which of these women have pre-neoplastic disease. In order to identify those women with lesions that need to be referred for further diagnosis, triage methods are used. Many countries use cytology for triage, including Argentina, and it is one of the recommended methods indicated by WHO guidelines [5]. Cytology as triage after HPV-test is more sensitive than cytology read without knowledge of the HPV status [6]. In this protocol, women who are HPV+ with abnormal cytology are referred for further diagnosis and treatment as needed, and HPV-negative women are generally recommended re-screening at 3–5 years. In Argentina, national guidelines decided by agreement with scientific societies that rescreening is recommended at 5 years [7]. For the sub-group of women who are HPV+/ cytology negative the recommendation is to repeat the HPV-test within 12 to 18 months [8]. Many of these infections are transient and will regress spontaneously, hence referring these women for colposcopy would imply overburdening diagnostic services with women whose risk of CIN2+ appear to be low [7] and it might also cause unnecessary psychological distress.

Assuring high levels of re-test among these women is particularly important, as studies have shown that they are at increased risk of CIN2+, even when the repeat HPV test is negative [8, 9]. Re-testing them at 12–18 months allows identifying persistent infections. In addition, there is also a problem linked to the moderate cytology sensitivity, which might result in CIN2+ lesions being missed by triage; sensitivity of the triage PAP is increased when women are retested.

However, despite the importance of following-up this group, evidence indicates that assuring high levels of re-test among these women is difficult to achieve [10], especially in settings where no call/recall systems are in place [11].

Evidence from cytology-based programs has shown that in general, completion of follow-up steps and adherence to recommended timeframes is especially difficult to achieve for women with low socio-economic level, as well as for those who face geographic, and health system barriers to health care [12–16]. These factors that affect follow-up in cytology-based programs might also be affecting re-test of HPV+/cytology negative women, however, there might also be factors derived from the HPV-testing context.

As today, there is no study assessing factors that might be associated with lower rate of re-test among HPV+/ cytology negative women in programmatic context in low-middle income populations. In Argentina HPV-testing was first introduced through the Jujuy Demonstration Project (JDP), during 2012–2014 [14] . We analyzed data from the JDP to identify those factors that might be associated with lack of re-testing among HPV+/cytology negative women.

## Methods

### Materials

We analyzed data collected for the JDP, carried out between 2012 and 2014 [14]. The JDP has been extensively described elsewhere [14, 15], but succinctly it introduced HPV-testing as primary screening for women aged 30 years and older attending the public health system. The programmatic target population was 30–64 years old, however screening was also provided to women aged 65 and over if requested. HPV samples were collected by health professionals at health centers, and since 2014 HPV self-collection was introduced as a programmatic strategy to increase screening coverage [15, 16]. Based on results of the JDP, HPV-testing was incorporated as primary screening at country level. The type of screening was mainly opportunistic, except for those women using self-collected method, who were reached by health workers during home visits using a nominalized list. Women were informed of their results at health care centers. Appointments for repeating the test were arranged at this visit in case of HPV+/cytology negative results.

Data of the JDP was registered in the National Screening Information System, (SITAM, by its initials in Spanish) implemented by the National Cervical Cancer Prevention Program (NCCPP). SITAM is a unified database that collects data about screening, diagnosis and treatment of all women attending the public health system. Those that were initially screened by the public sector but continued their follow-up in private services were recorded in SITAM only upon confirmation by the provincial prevention program; otherwise they were considered as loss to follow-up. For this analysis, we included women aged 30 years and older HPV-tested between 2012 and 2014 for the first screening test and data until 2018 for their re-test. We do not consider second tests that were done after a five-year period. Detailed description of how data is collected by the NCCPP is described elsewhere [14, 16]. The data are accessed by authorized healthcare workers and researchers. A non-disclosure agreement of the personal data is signed before a user and password is provided to access to the databases.

### Independent variables and outcome

Independent variables considered for this study were age, health insurance status, year of HPV-testing, record of previous Pap, HPV-testing modality (clinician-collected (CC) tests/self-collected (SC) tests), and span between HPV-test collection and report of results. This last variable is included as a proxy measuring the laboratories’ time efficiency processing the samples.

Primary outcomes were re-test attendance, and re-test attendance within recommended timeframe, both recorded as dichotomous variables. Re-test was considered within the recommended time frame when was performed between month 12 and 18 after primary screening [17]. Women who returned up to 30 days off the range were also included.

### Statistical analysis

We performed a set of descriptive statistics and different logistic regression models.

First, a multivariable regression was used to examine the association with re-test attendance of the following variables: age, health insurance status, year of HPV-testing, record of a previous Pap test, HPV-testing modality, and span in days between HPV-test collection and when result report was available in the system. We report odds ratios with 95% confidence intervals and *p*-values.

Secondly, we analyzed factors associated with re-test attendance within the recommended timeframe as outcome. Last, we performed a logistic regression including only women with re-screening test to assess the association of the covariates and the outcome within this group. This model worked as a sensitivity analysis model that helped to evaluate the association of the covariates and the outcome in this subset of women.

In order to explore possible interactions between the covariates, we tested different regressions including interaction terms for each pair of variables. Furthermore, we ran a model stratifying the data by year to confirm the associations for each year data. R Statistical software and R-Studio were used to perform the analysis.

## Results

**Fig. 1:**
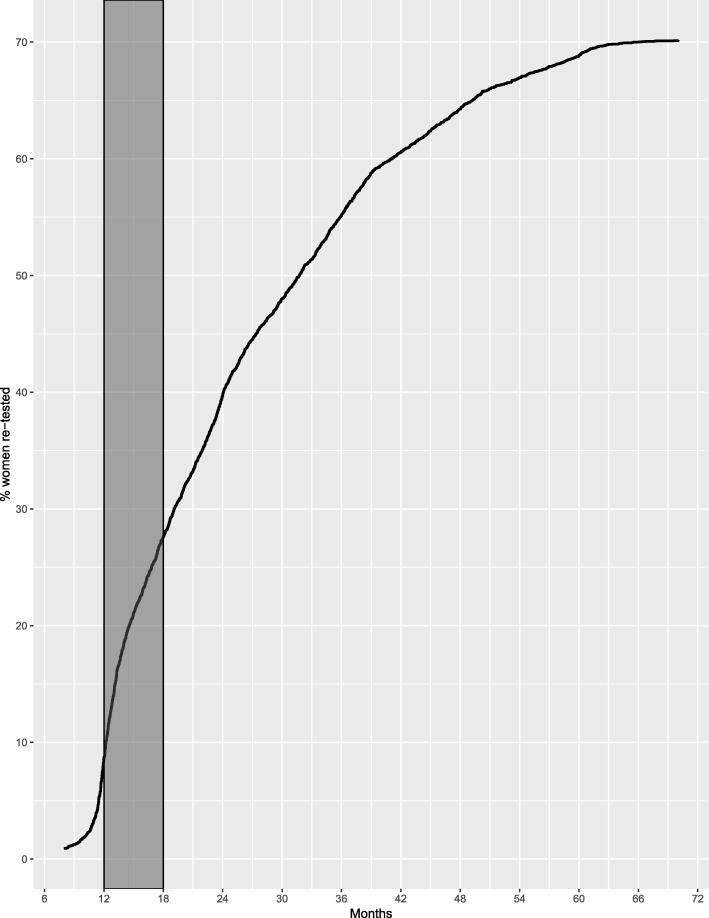
Percentage of women with a HPV re-test over time

For the period 2012–2014, 49,565 women were tested using HPV-test as primary screening test. Socio-demographic characteristics of these women are presented in Table 1. Of the total screened women, 6742 had a positive HPV-test, of which 4522 were HPV+/Cytology negative (67.1%). In total, 3172 HPV+/Cytology negative women (70.1%) have a record of a second HPV test as of March 2018. When the recommended timeframe is taken into consideration, 1196 women (26%) attended for re-screening at months 12–18 (Fig. 1). The median time for re-screening was 656.5 days, being the first quartile 411.2 and the third quartile 1015 days. The results for the second HPV test were positive for 1412 women (44.5%) and negative for 1759 (55.5%).

**Table 1:** Socio-demographic characteristics of HPV+/Cytology- women. Jujuy

	With HPV re-test		Without HPV re-test	
	n	%	n	%
Age				
Mean (Years)	40.16	SD (9.61)	41.61	SD (10.99)
30–34	1193	36.6	461	34.15
35–44	1115	34.8	457	33.85
45–54	512	16.4	231	17.11
55–64	290	9.4	136	10.07
65+	59	2.7	64	4.74
Previous PAP				
No	1657	52.24	971	71.93
Yes	1515	47.76	379	28.07
Health Insurance				
Public	2143	67.56	869	64.37
Private	1026	32.35	478	35.41
HPV-testing modality				
Self-Collected	125	3.94	133	9.85
Clinician-Collected	3047	96.06	1217	90.15
Span to re-test				
Within range	1196	37.62		
Outside range	1983	62.38

Results of the multivariate regression are shown in Table 2. The odds of being re-screened was 0.46 times among women with no record of a previous Pap test when compared to those who do had it (OR:0.46, 95% CI: 0.4–0.53, *p* < 0.001). Moreover, those women aged 64+ were also less likely to be re-screened, having 0.46 times the odds of being re-screened when compared to those aged 30–34 (95% CI: 0.31-0.68, *p* < 0.001). Women with CC tests had 1.42 times the odds of being rescreened compared to those with SC HPV-tests (95% CI: 1.06–1.91, *p* < 0.001). Women HPV-tested during 2013 and 2014 were less likely to be re-screened (OR: 0.65, 0.48; 95% CI: 0.56-0.76, 0.4–0.58, respectively with *p* < 0.001). Stratifying by year, results are consistent, being age, record of a previous Pap test, and HPV-test collection modality significant predictors. No significant interaction was found in the model testing all the covariates pairs.

**Table 2:** Multivariate logistic regression. Variables associated with having an HPV re-test. Jujuy Province, Argentina

	OR (95% CI)	OR (95% CI)
	With HPV re-test regardless date	With HPV re-test between12 to 18 months
Age (years)		
30–34	ref.	ref.
35–44	0.98 (0.84, 1.15)	0.91 (0.77, 1.08)
45–54	0.87 (0.72, 1.07)	1.01 (0.82, 1.25)
55–64	0.83 (0.65, 1.05)	0.95 (0.72, 1.23)
65+	0.46^***^ (0.31, 0.68)	1.36 (0.79, 2.32)
First HPV-test year		
2012	ref.	ref.
2013	0.65^***^ (0.56, 0.76)	0.97 (0.83, 1.14)
2014	0.48^***^ (0.40, 0.58)	1.13 (0.92, 1.40)
Health insurance		
Private	ref.	ref.
Public	1.01 (0.88, 1.17)	1.10 (0.94, 1.29)
Record of previous PAP		
Yes	ref.	ref.
No	0.46^***^ (0.40, 0.53)	0.82 ^*^(0.71, 0.95)
Time from sample taking to report emission		
Less than a month	ref.	ref.
More than a month	0.84 (0.62, 1.14)	1.10 (0.78, 1.53)
HPV-testing modality		
Self-collected	ref.	ref.
Clinician-collected	1.42^**^ (1.06, 1.91)	2.10^***^ (1.36, 3.32)
Constant	4.09^***^ (2.82, 5.95)	0.31^***^ (0.18, 0.50)

*OR* odds ratio, *CI* confidence interval

*p*-values: 0 ‘***’ 0.001 ‘**’ 0.01 ‘*’ 0.05 ‘.’ 0.1 ‘ ’ 1

When the outcome considered for the model was re-screening within the recommended time frame, being CC HPV-tested increased 2.10 times the odds of re-screening within 12–18 months (95%, CI: 1.36–3.32, *p* < 0.001), while women with no record of a previous Pap test were less likely to be-rescreened within 12–18 months (OR: 0.82, 95%, CI:0.71–0.95, *p* = 0.02).

Among the subset of re-screened women, the odds of re-screening within the recommended timeframe were reduced for those without record of a previous Pap test (OR: 0.84, 95% CI: 0.72–0.96, *p* = 0.016). On the contrary, women who had CC HPV-testing were more likely to be re-screened within the recommended timeframe. (OR: 2.82, 95% CI: 1.70–4.82, *p* < 0.001). The other covariates showed no statistically significant results.

## Discussion

This is the first study analyzing factors associated with attendance to re-testing by HPV+/cytology negative women using a large database of a low-middle income population where HPV testing is the primary screening method.

Screened women with an HPV +/ cytology negative result is a group that did not exist in cytology-based programs. In settings using HPV-testing and cytology for triage most HPV+ women will have normal triage cytology as result and will need re-screening at 12–18 months. Analyzing this subset of women in a separate way is important, since their overall risk of developing cervical cancer is increased [8]. They might also be facing specific barriers to follow-up, if for example they do not understand the meaning of the test/triage results. Thus HPV+/cytology negative women might behave differently regarding healthcare decisions [18, 19].

Our study found that HPV+/ cytology negative women with no previous record of a Pap test, aged 65+ and with SC tests are less likely to be re-tested. If the 12–18 months period for re-test is considered, women with SC-tests and with no Pap smears in the past have a decreased probability of being retested.

In our study, the overall rate of re-screening was 70.1%, close to what has been found by the POBASCAM study in Amsterdam, where the re-testing rate was 77%, and by Rijkaart et al. who reported a rescreening rate of 60% [10]. Pasquale et al. [20] and Passamonti et al. [21] reported -in well-organized programs in Italy- a higher percentage of HPV+/cytology negative women being retested (84%). However, in our study, if the 12–18 month recommendation is considered, the re-screening rate drops significantly, as only 26% of re-screened women did the test within this timeframe. The decreased percentage of women attending to re-testing within recommended timeframes has been reported in high income-countries as well. A study in Denmark showed that 58% of women being retested attended within 18 months [22]. Low re-testing rates within recommended timeframes has also been a problem in cytology-based programs. In the US, about 20% of women eligible for cervical cancer screening have not been reached by screening programs within the timeframe recommended [23, 24]. These studies show that low-income groups, recent immigrants, and ethnic minorities have the lowest follow-up rates. [25] Other studies carried out in other high income countries have found similar disparities [26].

Factors associated to the loss to follow-up in cervical cancer prevention have been generally discussed, however, there is almost no information published about specific factors associated to loss to follow-up in the group of HPV+/cytology negative women [27]. The variables our study found associated to loss to follow-up were mainly related to the prior screening history, age and type of screening modality. These factors has been described as proxies for reduced access to health services [1, 15].

Studies that analyzed why screened women with positive results failed to complete follow-up and treatment in America Latina found that in most cases the reasons were related to a deficient health services organization and subjective factors [18, 28–31]. For example, in a study carried out in Jujuy before HPV-testing was introduced, delays in result delivery or not receiving results at all was the most commonly reported problem by women, followed by problems with appointment dates, and long waiting times [28]. In addition, in that study, 30% of the women reported subjective reasons, including fear, unwillingness to continue with the treatment, lack of proper information about the disease [28].

Luque et al. [32] found that age, marital status, and number of previous medical office visits were factors associated with adherence to the recommendations in the US. Rendle et al. [19] found that among HPV negative women from northern California HPV screening program, African American, Hispanic and, American Indian were more likely to be rescreened passed the interval recommended. Although these studies were performed in a high-income country, age and number of previous medical office visits are similar to those we found in our study.

In our study, women aged 65+ were less likely to be re-screened. Beavis (2017) and Li (2017) found that women in this age group have a higher rate of HPV persistent infection and a higher incidence of cervical cancer [33, 34]. Furthermore, Skaznik-Wikiel et al. (2012) concludes that the incidence of cervical cancer does not decrease significantly in older women and that women aged 70+ are frequently diagnosed when the disease has reached advanced stages, reducing treatment options [35]. This underscore the importance for prevention programs to enhance follow-up strategies focused on this age group.

Our study also found significant reduced odds of attending to rescreening among those women with no previous record of a Pap. In line with what other authors discussed, this factor seems to be linked to persistent socio-economic and geographic barriers to access health services, as well as the inadequacy of the health system to provide required services, indicating not only a barrier in the past, but a lasting obstacle to for appropriate follow-up [14, 31].

Moreover, women with CC HPV-Tests were at increased odds of re-screening. Women offered SC tests in the JDP were socially vulnerable women, with reduced access to screening [16]. Arrossi et al. (2016) described that one main reason why women choose HPV SC-tests is because it simplifies their health care process and reduce barriers such as responsibility for domestic work, work and family organization issues, and troubles navigating health care services’ organization [36]. Therefore, while self-collection highly increased their access to screening [15], social and health system barriers might still be operating in the continuation of the follow-up process. In addition, women screened at health centers might also have increased access to information about what are the implications of an HPV +/ Cytology – result from health workers and professionals.

There are different strategies described for decreasing the number of lost to follow-up in cervical cancer screening. Referral to colposcopy of these women implies a significant burden for the health system that will not be translated in better screening outcomes [17]. Setting up specific strategies to overcome barriers in the access to health services, such as improving the availability of appointments, as well as the implementation of effective technical and logistical changes on how to deliver the results has been pointed out as solutions to improve the adherence rates [37]. The implementation of mHealth interventions in this settings are currently being evaluated as an strategy to reduce lost to follow-up [38].

Notwithstanding the useful information provided by this study for future research on HPV+/Cytology negative women, and the contribution for further programmatic evaluations, a number of limitations exist. First, the population analyzed is that targeted and reached by the NCCPP, which might not be representative of the whole population. Also, the variables observed were those that the program collects as routine, other significant variables were not possible to include in the analysis. Further studies are needed to scale up these conclusions. Second, the nature of this study is observational, and it is not conclusive evidence of causal relationship between the variables analyzed and the outcome. Moreover, while in randomized study designs it is expected that unobserved confounders are randomly distributed among the groups without affecting the association between exposure and outcome, in this study -an observational study- unobserved confounder might be biasing the results.

Furthermore, the lost to follow-up is also a limitation for this study, since women that opt to continue their follow-up using private health providers are not necessarily included in SITAM.

## Conclusion

The transition to HPV test-based screening programs has defined a new group of women, those with an HPV +/normal cytology result. Low re-testing and low adherence to protocol recommendations have been found in this population. Our study found three factors associated to low attendance to re-screening: women without record of a previous Pap test, being aged 64+ and being screened with SC-tests. Further studies are needed to properly assess the risk of this group of women, and specific interventions might be needed to increase the effectiveness of the screening programs among HPV+/Cytology negative women. Particularly, among the groups that this study found associated to higher lost to follow-up, setting tailored measures, such as the reduction of barriers in the access and the implementation of logistics changes on how to deliver the results should be considered for future interventions.

## Abbreviations

CC: Clinician-collected tests
CI: Confidence interval
CIN2: Cervical intraepithelial neoplasia
HPV: Human Papillomavirus
JDP: Jujuy Demonstration Project
NCCPP: National Program on Cervical cancer Prevention Program
OR: Odds Ratio
SC: Self-collected tests
SITAM: National Screening Information System (by its initials in Spanish).
WHO: World Health Organization
